# The effects of crocin on spatial memory impairment induced by hyoscine: Role of NMDA, AMPA, ERK, and CaMKII proteins in rat hippocampus 

**DOI:** 10.22038/ijbms.2019.30138.7266

**Published:** 2019-06

**Authors:** Maliheh Adabizadeh, Soghra Mehri, Mahshid Rajabpour, Khalil Abnous, Marzieh Rashedinia, Hossein Hosseinzadeh

**Affiliations:** 1 Department of Pharmacodynamy and Toxicology, School of Pharmacy, Mashhad University of Medical Sciences, Mashhad, Iran; 2 Pharmaceutical Research Center, Pharmaceutical Technology Institute, Mashhad University of Medical Sciences, Mashhad, Iran; 3 Department of Pharmacology and Toxicology, Faculty of Pharmacy, Shiraz University of Medical Sciences, Shiraz Iran

**Keywords:** AMPA, CaMKII, Crocin, ERK, Memory, NMDA, Saffron

## Abstract

**Objective(s):**

*Crocus sativus* L. and its active constituent, crocin, have neuroprotective effects. The effects of crocin on memory impairment have been mentioned in studies but the signaling pathways have not been evaluated. Therefore, the aim of this study was to evaluate the effects of crocin on the hyoscine-induced memory impairment in rat. Additionally, the level of NMDA (N-methyl-D-aspartate receptors), AMPA (α-amino-3-hydroxy-5-methyl-4-isoxazole-propionicd acid), ERK (extracellular signal-regulated kinases), CaMKII (calcium (Ca^2+^)/calmodulin (CaM)-dependent kinaseII) mRNA and proteins were determined in rat hippocampus.

**Materials and Methods:**

Crocin (10, 20, and 40 mg/kg), hyoscine (1.5 mg/kg), normal saline and rivastigmine were administered intraperitoneally to male Wistar rats for 5 days. The effects on memory improvement were studied using Morris water maze (MWM) test. Then, the protein levels of NMDA, AMPA, ERK, pERK, CaMKII and p.CaMKII in hippocampus were analized using the Western blot test. Furthermore, the mRNA levels of NMDA, AMPA, ERK and pCaMKII genes were evaluated using real-time quantitative reverse transcription-polymerase chain reaction (qRT- PCR) method.

**Results:**

Aadminestration of crocin (20 mg/kg) and rivastigmine significantly improved learning and memory impairment induced by hyoscine. Also, administration of hyoscine reduced protein level of pERK, while treatment with crocin (20 mg/kg) recovered the protein level. No changes were observed in the protein levels and mRNA gene expression of NMDA, AMPA, ERK, CaMKII and pCaMKII following adminestration of hyoscine or crocin.

**Conclusion:**

Adminestration of crocin improved memory and learning. The effect of crocin in this model can be mediated by alteration in pERK protein level in rat hippocampus.

## Introduction

Memory is the ability to keep learning information (1) and relies on the activity of several brain constructions (2, 3). Among the different constructions of brain, which are involved in memory formation, hippocampus is an important structure (2, 4, 5). There are two types of memory including short-term and long-term memory (2, 3). The synaptic plasticity and modification of synaptic strength in the brain are necessary for learning and memory process (6).

Synaptic plasticity has two forms including long-term potentiation (LTP) and long-term depression (LTD) (7) which are modulated by several neurotransmitters, multiple receptors and postsynaptic signal transduction pathways (2, 4, 8).

The synthesis of new proteins which plays a pivotal role in LTP (9, 10) requires temporary alteration in the intracellular signaling pathways (11). 

Acetylcholine and glutamate have a significant function in learning and memory activities in the hippocampus (12, 13).

After release of glutamate from presynaptic membrane, α-amino-3-hydroxy-5-methyl-4-isoxazole-propionicd acid (AMPAR) and N-methyl-D-aspartate receptors (NMDAR) are activated, then intracellular calcium content, one of the serious intracellular second messenger for the induction of LTP, is elevated (14, 15). 

Other important ways for enhancement of intracellular calcium are the voltage-gated calcium channel (VGCC) and metabotropic glutamate receptor (mGluR) (14, 16, 17) . 

Calcium can activate calcium (Ca^2+^)/Calmodulin (CaM)-dependent kinase (CaMK) and protein kinases A and C, then leads to mitogen-activated protein kinase (MAPK) activation. There are three subsets of MAPK : c-Jun NH2-terminal kinase (JNK), p38 MAPK, and extracellular signal-regulated kinase (ERK) (18). In addition, MAPK can be conveyed to the nucleus and activate CAMP response element-binding protein (CREB) and consequently the expression of downstream genes such as tissue-type plasminogen activator (tPA) and brain-derived neurotrophic factor (BDNF) are changed (14, 15). 

In addition, when acetylcholine is released from presynaptic membrane, it can stimulate mAChRs and nAChRs and make status desirable for neuronal plasticity and activate various intracellular transduction pathways containing the ERK pathway (2, 19).

mAChRs are placed in presynaptic and postsynaptic membranes overall the brain. Presynaptic mAChRs can work as inhibitory autoreceptors upon cholinergic terminals and decrease glutamate release from synapses (20). Some studies have been shown sustained low-level activation of NMDAR with glutamate are fundamental in memory process (21).

Some studies have been exhibited that specified plants can be useful for cognitive disturbances (22). Several plants such as *Hypericum perforatum*, *Ginkgo* *biloba *and *Panax ginseng* showed considerable effects on memory and learning (23-26).

*Crocus sativus* L. (saffron) belongs to the Iridacea family (27). There are more than 150 components in stigma of *C. sativus* (28) and the most important and pharmacological active constituents in stigma are crocin, picrocrocin and safranal (27, 29, 30).

Crocin is a water soluble compound and is responsible for red color of saffron (31). Crocin has exhibited a wide range of biological effects including antioxidant (32, 33), antitumor (34, 35), anti-inflammatory (36), antidepressant (37-40), anticonvulsant (41, 42), sexual activity enhancer (43), antilithiasis (44), anti-obesity (45), genoprotective (46), and memory enhancer effects (47, 48). 

Some studies have been reported the efficacy of crocin on memory improvement (49, 50). It has been reported that chronic stress increased the level of glucocorticoids in body (51) and high the level of glucocorticoids caused oxidative damage in hippocampus of rats (52). Consequently, oxidative damage impaired memory function while administration of crocin reversed this impairment (53). In another study, crocin significantly ameliorated memory dysfunction induced with chronic cerebral hypoperfusion in rats (54).

However, the effects of crocin on memory dysfunction in some models have been reported but the exact mechanism and signaling pathways, which are involved, are not studied. Therefore, in the current study, the effects of crocin on memory impairment induced by hyoscine were evaluated in rats. Additionally, the levels of proteins including NMDA, AMPA, ERK, pERK, CaMKII, pCaMKII as important mediators in memory and learning phenomena were determined. 

## Materials and Methods

***Chemicals***

Hyoscine and rivastigmine were obtained from Cipla (India). Ethylene glycol tetra acetic acid (EGTA), ethylene diamine tetra acetic acid (EDTA) and protease cocktail inhibitor were purchased from Sigma-Aldric (Germany). N,N,N’,N’-Tetra methyl ethylene diamine (TEMED) and sodium dodecyl sulfate (SDS) were prepared from Merck, (Germany). Polyvinylidene fluoride (PVDF) membrane was obtained from Bio Rad (USA). 

***Crocin preparation***

The stigmas of *C. sativus* L. were obtained from Novin Saffron (collected from Ghaen, Khorasan province, Northeast of Iran). The extraction and purification of crocin were done according to the method as previously described (55). The purity of crocin was 97%.

***Animals***

Male Wistar rats, 220 ± 30 g were housed in colony rooms with 12/12 hr light/dark cycle at 21 ± 2 ^°^C and had free access to food and water. Animal experiments were done according to Mashhad University of Medical Sciences, Ethical committee Acts (910450).

***Experimental groups***

In this study, 36 rats divided at random in 6 groups (n=6) and the treatment of animals was carried out as follows: 

1) Control, Normal saline, 2) Hyoscine, 1.5 mg/kg (56), 3) Rivastigmine, 2 mg/kg +Hyoscine, 1.5 mg/kg (56), 4) Crocin, 10 mg/kg+ Hyoscine, 1.5 mg/kg (57), 5) Crocin, 20 mg/kg+ Hyoscine, 1.5 mg/kg (57), 6) Crocin, 40 mg/kg+ Hyoscine, 1.5 mg/kg (57). 

In all of the groups, normal saline, hyoscine, rivastigmine or crocin were administrated intraperitoneally (IP) for 5 days.

***Morris water maze test***

In the present study, Morris water maze test was used to determine the effect of hyoscine and crocin on spatial memory of rats (memory impairment was induced by hyoscine in rats and crocin was used to improve memory). 

A tank with 136 cm in diameter was filled with water (20-22 ^°^C) and contained four quadrants and a platform with 10 cm in diameter was placed in southwest, 1 cm below the water level, which rats could not see it. The pathway of rats was recorded with a camera. 

Rats were trained for 5 days. In everyday, rats experienced four trials per day. In each trial, rats were allowed to swim freely for 60 sec to find the platform, if the rats could not find the platform, they were guided to the platform and after 30 sec, the next trial was started. Two days after the last training day, the probe trial was done. In this test, the platform was removed from the tank and rats were tested for 60 sec in each 4 trials in the probe test. During training days, the latency time for finding platform was recorded. In probe test, the time spent in the quadrant where the platform had been located during the training days was measured (58). 

***Western blot analysis***

For Western blot analysis, the hippocampi of rats were separated from the brain and were homogenized with homogenized buffer which containing Tris (49.9 mM), EDTA (18.9 mM), EGTA (1.99 mM), NaF (10 mM), sodium orthovanadate (0.978 mM), β glycerophosphate, sodium deoxycholate (4.82 mM), 2-mercapto ethanol and complete protease inhibitor cocktail. Then, homogenized tissues were sonicated and centrifuged (10000 g, 4 ^°^C) respectively for 40 sec and 10 min. The total proteins were separated in 8-12% SDS-PAGE gels and transferred to PVDF membranes. Membranes were blocked with 5% skim milk or 5% bovin serum albumin (BSA) for different times at room temperature (ERK protein: 3 hr, CaMKII protein: 2 hr, AMPA protein: 2 hr, NMDA protein: 2 hr in skim milk and pERK protein: 1 hr, pCaMKII protein: 2 hr in BSA). After blocking, membranes were washed 3 times with 0.1% Tween 20 and TBS and incubated with primary antibodies (dilution ratio 1/1000) for 2 hr in room temperature (blocking with pERK antibody was done overnight in refrigerator). Primary antibodies were including: NMDA2A (Cell Signaling, # 4205S), AMPA (Cell Signaling, #2460S), CaMKII (Cell Signaling, #3362), pCaMKII (Cell Signaling, #3361), ERK (Cell Signaling, #9102), pERK (Cell Signaling, #9106). After blocking with primary antibodies, membranes were washed 3 times with 0.1% Tween 20 and TBS and after that were incubated with horseradish-peroxidase conjugated anti-rabbit antibody (Cell Signaling, #7074) or anti-mouse antibody (Cell Signaling, #7076) with 1:3000 dilutions for 1 hr at room temperature. Finally, enhanced chemiluminescnces (ECL) reagent and Alliance 4.7 Geldoc were used to detect protein bands. Protein bands were analysed using UVtec software (UK). The protein levels were normalized against beta actin intensity.

***Isolation of RNA and quantitative real time PCR***

The total cellular RNA was extracted from hippocampus tissue using TriPure Isolation Reagent kit (Roche, cat# 11667157001). The quality and quantity of total RNA were determined using Nano Drop (Thermo Scientific Nano Drop 2000 Uv-Vis, USA) spectrophotometer. For evaluating the expression of RNA, Express one-step SYBR GreenER^TM ^Kit (Invitrogen, USA) was used. According to the protocol of kit, primers and other material were mixed in a sterile microtube. Primers for this study were NMDA: (Forward: 5’-TGTCGGAAGTTCGTCAAG-3’; Reverse: 5’-CCAGGTAGAGGTCATAGGT-3’), AMPA: (Forward:5’-GAATCAGAACGCCTCAACGC-3’; Reverse: 5’-GCTCCGCTCTCCTTGAACTT-3’), ERK: (Forward: 5’-

AGAGTGGCTATCAAGAAG-3‘; Reverse: 5’-AGGTCCTGAA-

CAATGTAA-3’), CaMK: (Forward: 5’-GAATCTGCCGTCTCTTGA-3’;

Reverse: 5’-TTCTCTTGCCACTATGTCTT-3’), Β-actin: (Forward: 5’- GGGAAATCGTGCGTG ACATT-3 ‘; Reverse: 5’- GCGGCAGTGGCCATCTC-3’). These primers were selected using Beacon Design® software (Bio Soft, USA). After the preparation of samples the following one-step cycling programs were done for all genes: 5 min at 50 ^°^C for cDNA synthesis, 2 min at 95 ^°^C for PCR activation followed by 40 cycles of 15 sec at 95 ^°^C to denature the DNA and 1 min at 60 ^°^C to anneal and extend the templates. 

Then temperature gradually enhanced from 60 to 95 ^°^C and melting curve analysis was carried out. The specificity and efficacy of primers were determined respectively with standard and melting curves. β-Actin was used as internal control gene and data were analyzed using the ΔΔCT method. 

***Statistical analysis***

Results were expressed as means±SD. The comparison among groups was made using the One-way ANOVA followed by a Tukey-Kramer post test for unilateral comparison and Two-way ANOVA followed by a Bonferronis multiple comparison. *P*<0.05 was considered statistically significant. 

## Results

***The effect of crocin on memory impairment induced by hyoscine***

In the present study, the Morris water maze test was used to evaluate the effect of crocin with different doses (10, 20 and 40 mg/kg) on learning and memory impairments induced by hyoscine in rats.

**Figure 1:**
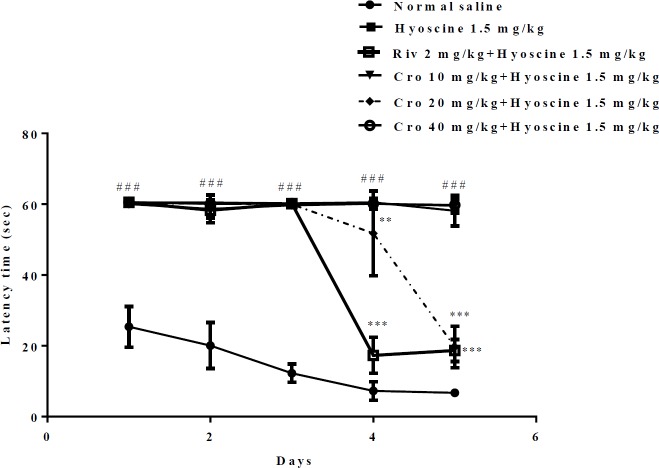
The percentage of time spent for finding the hidden platform during the training days (day 1-5). Data are expressed as the mean ± SD, (n=6). ### *P*<0.001vs. control, ***P<0.001 vs. hyoscine treated animals. Cro: crocin; Riv: rivastigmine

The results showed that hyoscine-treated animals spent more time for finding the hidden platform in comparison to control group. As shown in Figure 1 the latency time for finding platform in control group was 6.73 ± 0.92 sec on fifth day which significantly increased to 58.15 ± 4.3 sec in hyoscine received animals. 

Interestingly, administration of crocin markedly ameliorated memory dysfunction of hyoscine. During day one, two and three, crocin in different doses did not significantly change latency time when compared to hyoscine group. As exhibited in Figure 1 during day four and five crocin (20 mg/kg) improved memory function and animals found hidden platform quickly (*P*<0.01 and *P*<0.01 vs hyoscine in day four and five, respectively). 

Also, rivastigmine-treated animals exhibited memory improvement during day four and five compared to hyoscine group (*P*<0.001) (Figure 1). 

**Figure 2:**
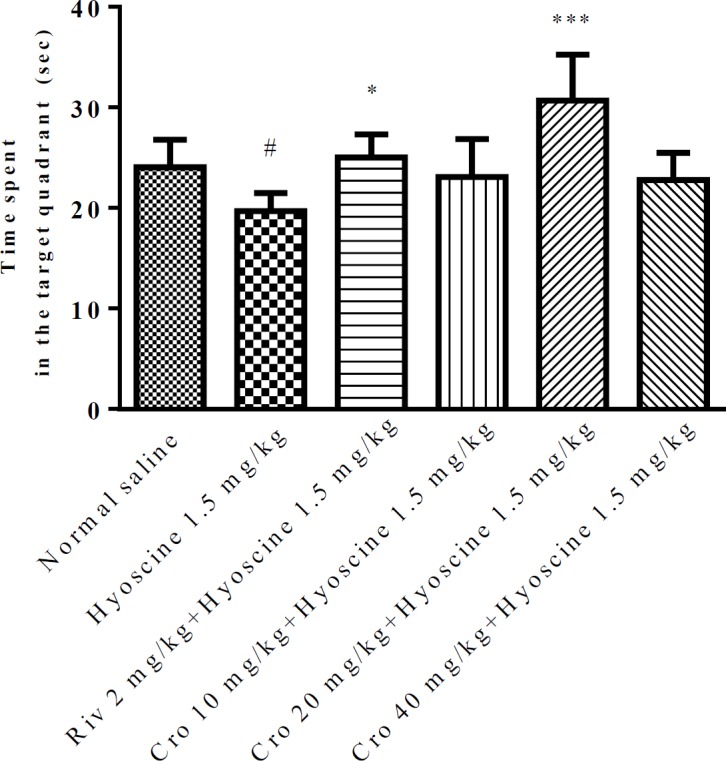
The percentage of time spent in target quadrant during the probe trial (day 8). Data are expressed as the mean±SD, (n=6). # *P*<0.05 vs. control, * *P*<0.05 and ***P<0.001 vs. hyoscine treated animals. Cro: crocin; Riv: rivastigmine

Additionally, the probe trial test was done on day eight and the time spent in target quadrant (Q_1_) was recorded for each rat. The results demonstrated that duration time in hyoscine group was significantly (*P*<0.05) lower than control group while crocin (20 mg/kg) significantly increased the presence of rats in Q_1_ when compared to hyoscine group (*P*<0.001) (Figure 2). Also, rivastigmine enhanced the attendance of rats in Q_1_ when compared to hyoscine group (*P*<0.05).

**Figure 3:**
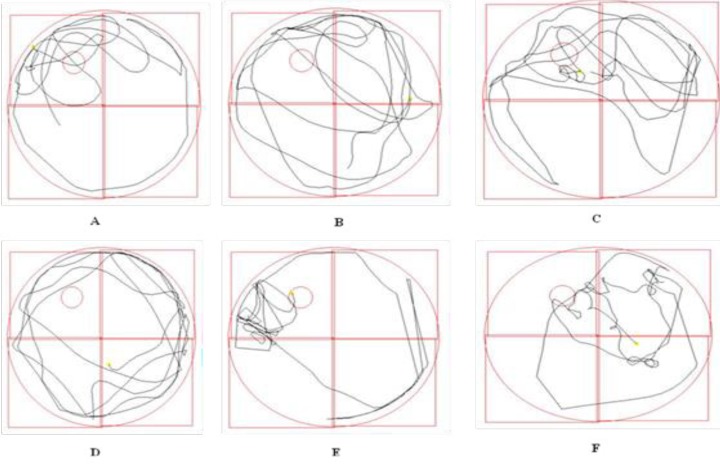
The representative pathway of rats in different groups in probe trial test

The path length of rats in probe trial was shown in Figure 3.

**Figure 4:**
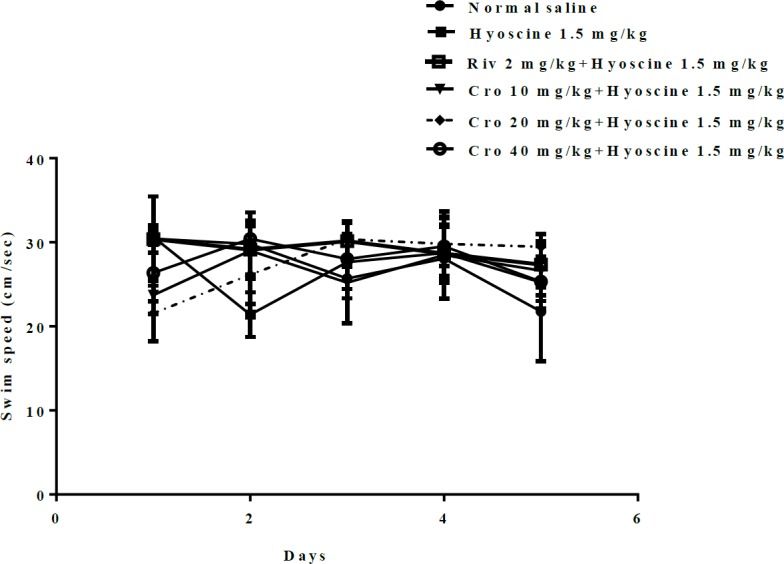
The effect of different doses of crocin (10, 20, 40 mg/kg) on speed of rats during training days. Cro: crocin, Riv: rivastigmine

**Figure 5:**
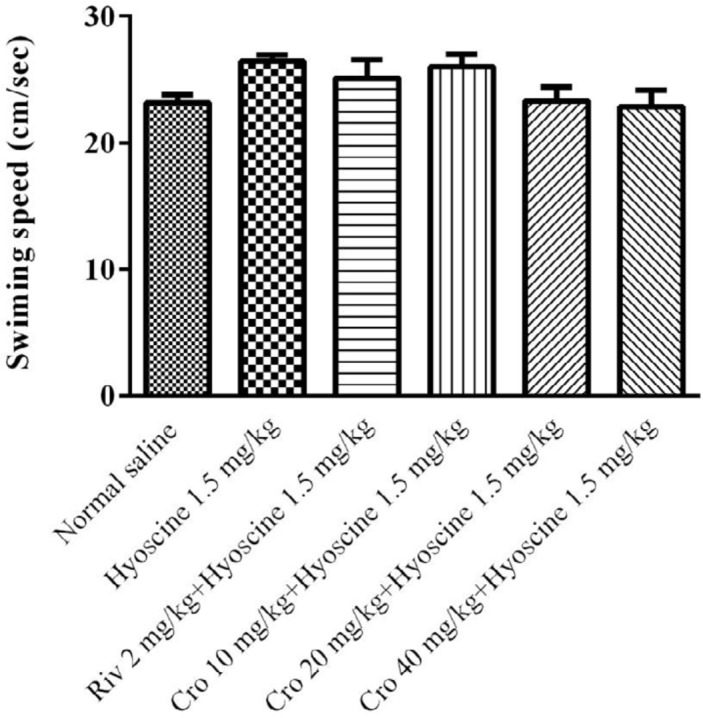
The effect of different doses of crocin (10, 20, 40 mg/kg) on speed of rats during probe trial day. Cro: crocin; Riv: rivastigmine

The effects of hyoscine and crocin (10, 20, 40 mg/kg) on locomotor activity of animals were evaluated. For this purpose, the swimming speed of rats during training days and probe trials were measured. There was no significant difference in swimming speed among experimental groups during training days (Figure 4) and probe trials (Figure 5).

***The effect of crocin and hyoscine on different proteins that involved in memory signaling*** ***pathways***

In this study, Western blotting analysis was used for evaluating the expression level of AMPA, NMDA, ERK, pERK, CaMKII, pCaMKII proteins that involved in memory improvement of crocin and memory impairment of hyoscine. 

**Figure 6:**
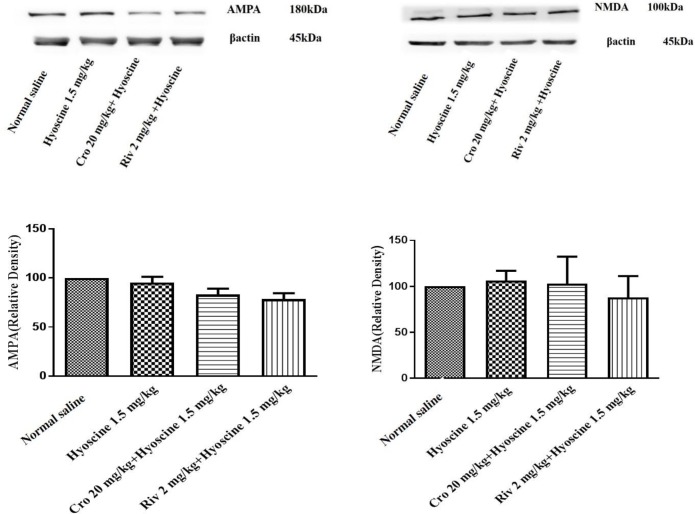
Effect of crocin (20 mg/kg) on NMDA and AMPA levels in hippocampus of rat following treatment with hyoscine. Data are expressed as the mean±SD, (n=4). Cro: crocin, Riv: rivastigmine

The results exhibited that the level of protein expression of NMDA and AMPA did not significantly change in different experimental groups (Figure 6). 

**Figure 7:**
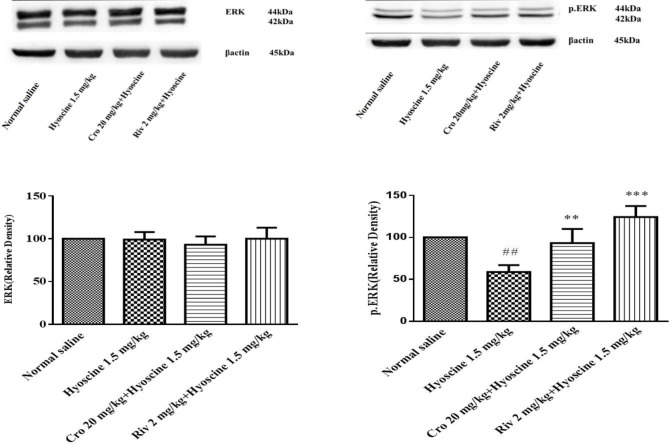
Effect of crocin (20 mg/kg) on ERK and pERK levels in hippocampus of rat following treatment with hyoscine. Data are expressed as the mean±SD, (n=4). ## *P*<0.01vs. control, ** *P*<0.01, ***P<0.001 vs. hyoscine treated animals. Cro: crocin; Riv: rivastigmine

**Figure 8:**
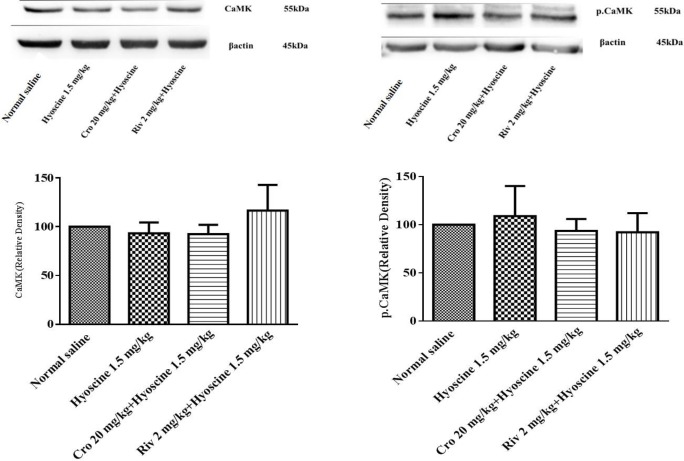
Effect of crocin (20 mg/kg) on CaMKII and pCaMKII levels in hippocampus of rat following treatment with hyoscine. Data are expressed as the mean±SD, (n=4). Cro: crocin; Riv: rivastigmine

Additionally no significant alteration in the level of CaMKII, pCaMKII and ERK proteins were observed following administration of rivastigmine, hyoscine and/ or crocin (Figure 7, 8).

Treatment with hyoscine resulted in a significant decrease of pERK protein level when compared to the control groups (*P*<0.01). Crocin (20 mg/kg) prevented this reduction in comparison to hyoscine received animals (*P*<0.01) (Figure 7). Also, rivastigmine could increase the level of this protein in comparison to hyoscine group (*P*<0.001, Figure 7).

***The effect of crocin on expression of different genes involved in memory signaling pathways***

**Figure 9:**
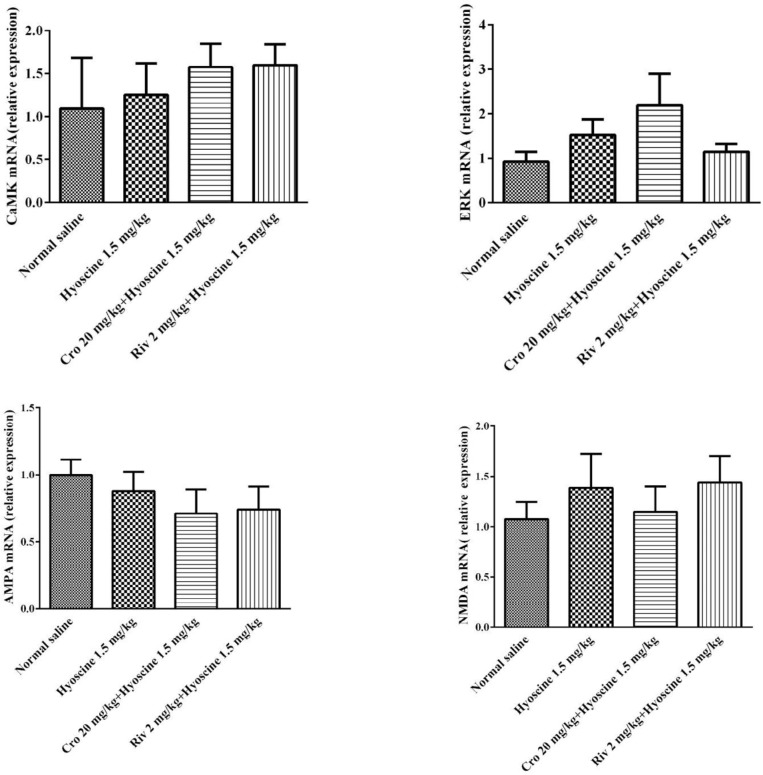
Effect of crocin and hyoscine on ERK, CaMKII, NMDA and AMPA mRNA expression in hippocampus of rats. mRNA levels were normalized against beta -actin. Data are expressed as the mean±SD, (n=4). Cro: crocin; Riv: rivastigmine

In this study, a quantitative real time- PCR was used to determine ERK, CaMKII, NMDA and AMPA mRNA expression. The administration of rivastigmine, hyoscine and/ or crocin did not change ERK, CaMKII, NMDA and AMPA RNA expression in different experimental groups (Figure 9). 

## Discussion

The results of this study showed that hyoscine impaired memory function in rats while administration of crocin (20 mg/kg) remarkably recovered memory dysfunction. In addition, according to acquired data, pERK protein level in hippocampus significantly elevated following treatment with crocin. 

Different studies showed that hyoscine, as an anticholinergic agent, could impair memory function (59-61) and could be mentioned as an experimental tool for evaluating the memory enhancement of different agents including herbal medicines (59, 61). 

Treatment with crocin recovered memory impairment which induced using chronic restraint stress (53) and brain ischemia (54). 

Although the memory enhancement effect of crocin has been shown previously but the underlying mechanisms have not been evaluated. Therefore, in the current study, the effects of crocin on hyoscine-induced memory impairment with focus on the expression level of AMPA, NMDA, ERK, pERK, CaMKII, pCaMKII proteins and related mRNAs were investigated. 

Results of this study represented that hyoscine impaired the memory of rats in the Morris water maze test, as the time to reach the hidden platform was enhanced during 5 days training in comparison to control groups. Crocin, 30 min prior to hyoscine administration, diminished the memory impairment of hyoscine from the fourth days of training. In addition, the results of probe trials day exhibited that rats receiving crocin (20 mg/kg) spent most of the swimming time in the target quadrant in comparison to hyoscine groups. These results demonstrated that crocin (20 mg/kg) group remembered the location of the platform better than hyoscine group, so crocin (20 mg/kg) can improve memory dysfunction induced by hyoscine.

A) Normal saline, B) Hyoscine, C) Rivastigmin, D) Crocin 10 mg/kg, E) Crocin 20 mg/kg, F) Crocin 40 mg/kg

Additionally, to evaluate the effects of hyoscine and crocin on locomotor activity, the swimming speed was determined. Interestingly, there was no significant different between treated groups. This represented the memory improvement of rats with crocin, was not due to the discrepancy in their locomotion activities.

At the next step, the molecular mechanisms, which are involved in memory improvement of crocin, were evaluated. The memory formation relies on the activity of neurotransmitter systems, several receptors, postsynaptic mechanisms and signal transduction pathways (2). In our study, the roles of NMDA and AMPA receptors, ERK, pERK, CaMKII and pCaMKII proteins in memory improvement effect of crocin were determined. 

Our results showed that pERK protein level decreased in hippocampus of rats following hyoscine administration when compared to the control group, while treatment with crocin significantly activated this protein. The levels of NMDA, AMPA, ERK, CaMKII and pCaMKII proteins were not significantly changed in experimental groups. Some studies exhibited disturbance in NMDA receptors impaired spatial learning (62, 63) and another studies demonstrated an important role for AMPA receptor in spatial learning (64). Intracellular Ca^2+^ ion binds to calmodulin and activated CaMKII can trigger MAPK signaling pathway. In addition, Ca^2+ ^can enter through the mGluR activation. Intracellular calcium can also activate pKA, CaMKII and MAPK (14). 

Similar to our study, the level of pERK protein was decreased following treatment with amyloid beta (Aβ) (65) and exposure to chronic intermittent hypoxia-hypercapnia (CIHH) (66) and consequently memory function markedly impaired. The elevation of pERK protein level significantly recovered memory impairment.

ERK protein can be activated with several neurotransmitters such as glutamate via metabotropic or ionotropic glutamate receptors, acetylcholine through muscarinic/nicotinic receptors and noradrenaline via β-adrenergic receptors. ERK protein is an important signal integrator in hippocampus long term and short term memories. It can induce synaptic plasticity and learning following activating cascade of intracellular processes (2). 

After activation of ERK, pERK dislocates to the nucleus, then it can alter gene expression and leading to the synthesis of novel proteins. cAMP response element-binding protein (CREB) and the Ets like gene-1 (Elk-1) are transcription factors that can be activate with ERK (2, 14). The effect of ERK on synaptic behavior and gene expression place this factor in a strategic position, leading crosstalk between different signaling pathways in formation both short term and long term memories (2).

According to our results, crocin administration significantly increased the level of pERK protein in the hippocampus of rat. This enhancement might be due to the effects of crocin on NMDAR and AMPAR signaling pathways in the hippocampus. Therefore, we evaluated the level of NMDAR and AMPAR to distinguish whether crocin impressed the NMDA and AMPA signaling pathways. Our results showed memory-enhancing effects of crocin in rat are not related to activation of the NMDA receptor pathway in the hippocampus, so it needs to evaluate another pathway that can activate the ERK signaling such as mGLU receptor of glutamate and β-adrenergic receptors of noradrenaline.

In our study, the mRNA levels of ERK did not correlate with the pERK protein levels. This poor correlation might be due to the various processes involved between transcription and translation such as transcriptional regulation and post-transcriptional regulation, which need further studies. 

## Conclusion

In conclusion, treatment with crocin strongly recovered hyoscine-induced memory dysfunction in rats. The effects of crocin in part can be due to the elevation of pERK protein level in rat hippocampus.
